# Essential Oil Composition and Bioactivities of *Waldheimia glabra* (Asteraceae) from Qinghai-Tibet Plateau

**DOI:** 10.3390/molecules22030460

**Published:** 2017-03-13

**Authors:** Ji De, Yan Lu, Lijun Ling, Nan Peng, Yang Zhong

**Affiliations:** 1 College of Science, Tibet University, Lhasa 8500, Tibet, China; 12110700018@fudan.edu.cn; 2 School of Pharmacy, Fudan University, Shanghai 201203, China; 13111030032@fudan.edu.cn; 3 School of Life Science, Fudan University, Shanghai 200438, China; nanpeng@fudan.edu.cn

**Keywords:** *Waldheimia glabra*, Tibetan medicine, essential oil, anti-influenza, anti-inflammatory

## Abstract

*Waldheimia glabra* is traditionally used as incense and as an anti-influenza drug by Tibetans in China. Here, we collected *W. glabra* from the Gangs Rinpoche mountain at an altitude of 5200 m, and analyzed its essential oil by gas chromatography-mass spectrometry (GC-MS) combined with the retention indices (RI). Twenty-seven compounds, representing 72.4% of the total essential oil, were identified, including α-bisabolol (20.2%), valeranone (11.8%), chamazulene (9.9%), spathulenol (8.2%), β-caryophyllene (6.1%), and caryophyllene oxide (5.2%). Bioactivity evaluation of the essential oil revealed that it exhibited potent anti-influenza effect on viruses H_3_N_2_ and anti-inflammatory effect by inhibiting the lipopolysaccharide (LPS)-induced nitric oxide (NO) production in RAW 264.7 macrophages, but no anti-complementary activity.

## 1. Introduction

*Waldheimia glabra* (Decne.) Regel (Tibetan name: ‘ghaan-poe’) is mainly distributed in India, Nepal, Pakistan, Afghanistan, and China. In China, *W. glabra* is mainly distributed in the areas of Tingri, Nyalam, Zhada, and Rituo at altitudes above 4000–5400 m in Tibet, along the Transhimalayan Mountain [1]. Himalaya is a global central region of species–rich diversity [2]. Many plants in this cold and high altitude region have been traditionally used as Tibetan medicine for their amazing efficacy in healthcare. Although it has not yet been recorded in Tibetan medicine, *W. glabra* was traditionally believed to prevent colds and fever by local people in Tibet by making incense or soup. As reported by Giorgi et al. [3] and Manzo et al. [4], *W. glabra* was used in Tibetan medicine for the treatment of influenza, cold, wounds, fever, and skin diseases. However, the bioactive constituents and pharmacological actions of this plant were rarely reported.

Plant essential oils always exhibit a number of biological properties, such as antibacterial, antiviral, anti-fungal, and anti-inflammatory activities, etc. [5]. The essential oil of *W. glabra* is supposed to have interesting bioactivities related to the medical use of *W. glabra*. As reported [4], 75–78 volatile compounds and 27 essential oil compounds in *W. glabra* from Khumbu Valley, Nepal have been determined using HS-SPME GC-MS [3] and GC-MS, respectively. However, depending on the method of extraction employed, their major constituents were quite different. The HS-SPME volatiles mainly contained α-pinene (38.87%–48.35%), β-phellandrene (25.86%), and seychellene (7.09%–11.54%) [3], while the essential oil mainly contained spathulenol (24.83%), α-bisabolol (7.80%), 9-tetradecenol (7.65%), thujopsene (6.60%), α-thujone (5.69%), and yomogi alcohol (5.08%) using IBM Statistical Package for Social Sciences v. 20 software (SPSS, IBM, Segrate, Milan, Italy) for statistical analyses [4].

Herein, we collected *W. glabra* from the Gangs Rinpoche Mountain at an altitude of 5200 m in Tibet (Trans-himalaya Mountain Range in Tibet), China and analyzed its essential oil constituents by GC-MS. In addition, for further exploitation of this plant, the possible bioactivities of the essential oil of *W. glabra* were examined, including anti-influenza, anti-inflammatory, and anti-complementary activities.

## 2. Results

### 2.1. Chemical Composition of Essential Oil

Hydrodistillation of *W. glabra* yield 1.5% (*v*/*w*) of the yellow essential oil using the Chinese Pharmacopoeia appendix method [6]. The identification by mass fragmentation and retention indices (RI) revealed the presence of 27 components representing 72.4% of the total oil. The components and their relative percentage are presented in Table 1. The results showed that the major constituents were α-bisabolol (20.2%), valeranone (11.8%), chamazulene (9.9%), spathulenol (8.2%), β-caryophyllene (6.1%), and caryophyllene oxide (5.2%), with oxygenated sesquiterpenes (59.1%) being the main class of compounds, followed by sesquiterpene hydrocarbons (8.7%) and oxygenated monoterpenes (3.9%), using relative quantification by GC-MS.

### 2.2. Anti-Influenza Activity of Essential Oil from W. glabra

In order to confirm the possible activity of *W. glabra* for anti-influenza in Tibetan folk medicine, the essential oil was evaluated for its cytotoxicity and inhibition against the influenza virus H_3_N_2_ in vitro by complete cytopathic effect (CPE) inhibition rate assay [7], with ribavirin as the positive control. As shown in Table 2, Ribavirin had good virucidal activity with IC_50_ value of 37.2 μg·mL^−1^ and no cytotoxicity at 250 μg·mL^−1^. The IC_50_ value of the essential oil was 88.8 μg·mL^−1^, suggesting the moderate anti-influenza effect of *W. glabra* essential oil. However, the essential oil showed cytotoxicity against the MDCK cells at 200 μg·mL^−1^, with the TC_50_ value of 252 μg·mL^−1^.

### 2.3. Anti-Inflammatory Activity of Essential Oil from W. glabra

**Figure 1:**
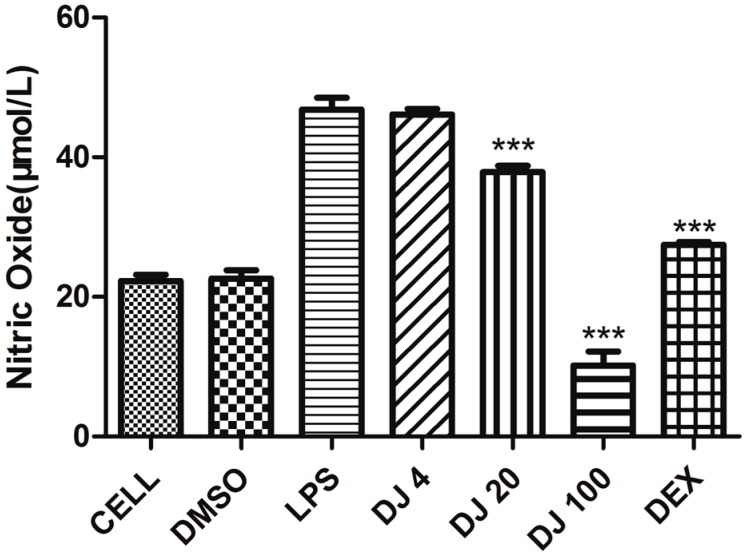
NO inhibitory effects of *W. glabra* essential oil on lipopolysaccharide (LPS)-activated RAW 264.7 cells. CELL: blank control; DMSO: blank control with DMSO; LPS: LPS control; DJ4, DJ20 and DJ100: the essential oil at 4, 20 and 100 μg·mL^−1^ respectively; DEX: positive control of Dexamethasone; ***: *p* < 0.001 vs. LPS group (*n* = 4).

Since the inhibitors of NO production in macrophages via lipopolysaccharide (LPS) stimulation are considered as anti-inflammatory agents, we determined the inhibitory effect of *W. glabra* essential oil on the NO production of RAW 264.7 macrophages using Griess assay [8]. As shown in Figure 1, the essential oil samples at 20 μg·mL^−1^ and 100 μg·mL^−1^ exhibited significant anti-inflammatory activities on the LPS stimulated cells in a dose-dependent manner. Especially, the essential oil at 100 μg·mL^−1^ showed a stronger inhibitory effect than the positive control.

### 2.4. Anti-Complementary Activity of Essential Oil 

The *W. glabra* essential oil was evaluated for in vitro anti-complementary activity on classical pathway with the method of Yin et al. [9]. The result indicated that the essential oil had no anti-complementary activity at the value of 50% inhibitory concentration (CH_50_) > 2 mg·mL^−1^.

## 3. Discussion

Tibetan medicine has been a hot topic in recent years because of its magical medicine theory, multi-herb compatibility complex system, and various medical effects. Therefore, the analysis of the chemical composition of this Tibetan medicinal herb and its bioactivities are of great importance for Tibetan medicine development. Herein, we firstly reported the in vitro anti-influenza and anti-inflammatory effects of the essential oil of *W. glabra*, which were concordant with the plant’s folk medicinal use on cold and fever in people. However, the essential oil did not show the expected anti-complementary activity. It is interesting that the non-volatile extract of *W. glabra* was found to show potent anti-complementary activity with CH_50_ less than 0.1 mg·mL^−1^ in the subsequent study, but no anti-influenza activity. It suggested that *W. glabra* is a very useful resource of Tibetan medicine to treat inflammatory diseases induced especially by the influenza virus, with the essential oil directly killing the influenza virus and the anti-complementary extract inhibiting the excessive activation of the immune system [9]. The different roles and synergistic effect of these two extracts on influenza virus induced inflammation and fever in vivo are worth investigating and discussing in the near future, as well as the possible mechanism. The major anti-influenza compounds of the essential oil and the anti-complementary constituents of the non-volatile extract will also be investigated.

The result of GC-MS analysis indicated that α-bisabolol, valeranone, spathulenol, caryophyllene, and caryophyllene oxide were the major compounds of *W. glabra.* It is worth noting that some major compounds had been isolated from other plants and reported to have various bioactivities. α-Bisabolol isolated from *Pogostemon speciosus* [10], *Eremanthus erythropappus* [11], and *Matricaria chamomilla* [12] was considered to have anti-inflammatory, antibiotic, anticancer, antimicrobial, and antioxidant activities. Valeranone in *Valeriana officinalis* [13] and *Valeriana wallichii* [14] were reported for its anti-inflammatory effect. Spathulenol in *Lantana camara* characterized antifungal activity [15]. Caryophyllene and caryophyllene oxide in *Teucrium pseudochamaepitys* presented antioxidant, cytotoxic, and antiviral activities [16]. Therefore, we can infer that the properties of anti-influenza and anti-inflammation of essential oil of *W. glabra* may due to these major chemical compounds. 

Many components of *W. glabra* essential oil collected in Tibet were similar to those reported from the Nepal sample [4], including α-pinene, yomogi alcohol, β-citronellol, artemisia alcohol, terpinen-4-ol, α-terpineol, caryophyllene, β-farnesene, β-chamigrene, (−)-aristolene, α-curcumene, H-2,4a-ethanonaphthalene, 1,3,4,5,6,7-hexahydro-2,5,5-trimethyl, aromadendrene, α-bisabolol, (+)-spathulenol, caryophyllene oxide, cedrol, hanphyllin, and spathulenol. However, there was a great difference in their major compounds and percent contents. The essential oil of *W. glabra* from Tibet, China mainly contained α-bisabolol (20.2%), valeranone (11.8%), chamazulene (9.9%), spathulenol (8.2%), β-caryophyllene (6.1%), and caryophyllene oxide (5.2%), whereas spathulenol (24.8%), α-bisabolol (7.8%), 9-tetradecenol (7.7%), thujopsene (6.6%), α-thujone (5.7%), and yomogi alcohol (5.1%) were reported as representative compounds in the essential oil of *W. glabra* from Nepal. 

The difference of aromatic compounds in the same species depends on different factors, such as extraction and analytical method, harvesting time, collecting parts, isolation procedure of aroma compounds, and climate change, etc. [17]. In addition, the Himalayas are a global central region of species-rich diversity, where slight changes on altitude and climate may influence the aromatic compounds [18]. On the other hand, the Himalaya Mountain region is considered as an important factor contributing to high population differentiation and a directional barrier to gene flow [19]. Therefore, the variation of chemical diversity in the same species in the Himalayan region is possible. The essential oils analyzed in this study and in the literature [4] were extracted by the method of distillation using the same aerial parts of *W. glabra*. However, the plant samples were collected at the end of August from Tibet and at the end of October from Nepal. The harvesting time and distribution might be the major factors for the difference of the volatile constituents in the two *W. glabra* samples.

## 4. Materials and Methods 

### 4.1. Plant Material and Extraction of Essential Oil 

The whole plant (flower, stems and leaves) of *W. glabra* was collected from the Gangs Rinpoche Mountain at an altitude of 5200 m, Qinghai-Tibetan Plateau, China at the end of August 2013, and identified by Prof. Peng-Cheng Lin (Qinghai Nationalities University, Xining, China). A voucher specimen (DJGb-2013730) was deposited in the Herbarium of Biodiversity Science and Geobiology of Tibet University. 

One hundred grams of the dried, crushed, whole plant of *W. glabra* were subjected to 1000 mL of distilled water, and then distilled according to the Chinese Pharmacopoeia appendix method [8], slightly boiled for 3 h, leading to 1.5 mL of the essential oil being collected.

### 4.2. Identification of the Components of Essential Oil

The GC-MS analysis of essential oil was carried out on a Thermofocus DSQII (Thermo, Wilmington, DE, USA), equipped with an HP-5MS column (30 m × 0.25 mm, film thickness 0.25 μm). The oven temperature was programmed from 60 °C (1 min isothermal) to 300 °C at a rate of 15 °C/min, and then kept for 8 min with a carrier gas at 1.0 mL/min. Both the temperatures of the MS transfer line and the injector were set at 250 °C. One microliter of essential oil sample was injected (in split mode 20:1). The mass spectra were obtained at 70 eV with a mass scan range of 41–400 amu. Compounds of essential oils were identified by matching their mass spectra with data bank mass spectra in NIST5.0 (National Institute of Standards and Technology), and comparing their retention to a homologous series of *n*-alkanes C_8_–C_30_ (Sigma-Aldrich, Steinheim, Germany) under the same operating conditions.

### 4.3. Anti-H_3_N_2_ Virucidal Activity

#### 4.3.1. Viruses, Cells, and Cell Culture

Influenza viruses H_3_N_2_ (A3/Beijing/30/95) was kindly provided by the Shanghai Municipal Center for Disease Control and Prevention (Shanghai, China).

The host cells of MDCK cells were provided by the Institute of Medicinal Biotechnology, Chinese Academy of Medical Sciences (Beijing, China). The cells were cultured in EMEM (Eagle’s minimum essential medium) containing 10% fetal bovine serum (FBS, Gibco, Life Technologies, Carlsbad, CA, USA), penicillin G (100 U/mL), and streptomycin (100 μg/mL). The cells were maintained in a tissue culture flask at 37 °C and in 5% humidified atmosphere of CO_2_.

#### 4.3.2. Cellular Toxicity Test 

MDCK cells with a density of 1 × 10^4^ cells per well were seeded into 96-well culture plates and incubated for 24 h until the cells reached 90% confluency. After incubation, the cells were treated with various concentrations of essential oil (200, 100, 50, 25, and 12.5 μg·mL^−1^) dilutions with RPMI-1640 medium containing 10% FBS, penicillin G (100 μg·mL^−1^), and streptomycin (100 μg·mL^−1^), respectively. These dilutions were incubated at 37 °C in 5% CO_2_ for 72 h. The cytotoxicity of the essential oil was determined by modified MTT assay, and cell viability was expressed with optical density.

#### 4.3.3. Determination of Anti-H_3_N_2_ Activity

The antiviral activity was assessed by CPE (inhibition of viral cytopathic effect) assay [7]*.* MDCK cells were seeded into 96-well culture plates and incubated for 24 h at 37 °C in humidified 5% CO_2_. Afterwards, cells were infected with influenza A H_3_N_2_ virus of 30 TCID_50_ and incubated for 2 h at 37 °C and in a humidified 5% CO_2_ atmosphere. Then, the supernatant was carefully removed. Subsequently, the cultures were treated with 100 μL *W. glabra* essential oil at different concentrations (200, 100, 50, 25, and 12.5 μg·mL^−1^) and with a positive control drug (ribavirin), respectively, and incubated for 72 h at 37 °C in a humidified 5% CO_2_ atmosphere. The CPE was observed to evaluate the antiviral activity of *W. glabra* essential oil, and IC_50_ (50% inhibitory concentration) was calculated.

### 4.4. Anti-Inflammatory

NO production was assessed according to the Griess reaction. Dexamethasone (DEX) was selected as a positive control. RAW 264.7 cells were provided by the Department of Pharmacology, School of Pharmacy, Fudan University, Shanghai, China. Each tested sample was dissolved in DMSO, and diluted with fresh FBS-free DMEM media to a final concentration with DMSO ≤ 0.02%. The RAW 264.7 macrophages were seeded in 96-well plates (2 × 10^5^ cells/mL) and co-incubated with samples (concentrations of 4, 20, and 100 μg·mL^−1^) and LPS (10 ng·mL^−1^)*.* After incubation at 37 °C for 24 h, the culture supernatant was mixed with 25 mL Griess reagent with (0.2% *N*-(1-naphtyl) ethylenediamine dihydrochloride and 25 μL of 2% sulphanilamide in 5% phosphoric acid solution) to determine NO production. Absorbance was measured at 570 nm using a microtiter plate reader.

### 4.5. Anti-Complementary Activity

Anti-complementary activation was assessed by the classical pathway based on the degree of hemolysis of erythrocytes. The various concentrations of essential oil of *W. glabra* were, respectively, dissolved in DMSO (dimethyl sulfoxide) and then diluted with barbital-buffered saline (BBS) (containing 0.1% gelatin, 0.5 mM Mg^2+^, 0.15 mM Ca^2+^, and pH 7.0). The DMSO final concentration was kept less than 1% to avoid interference with the complementary activities detection. Sensitized erythrocytes (EA) were prepared by incubating sheep erythrocytes (4.0 × 10^8^ cells/mL) with rabbit anti-sheep erythrocyte antibody. Various dilutions of tested samples (200 μL) were mixed with 200 μL of diluted guinea pig serum (1:80) and 200 μL EA. The mixture was incubated at 37 °C for 30 min and centrifuged for 5 min. The optical density of the supernatant (200 μL) was measured at 405 nm with a spectrophotometer.

## 5. Conclusions

In conclusion, the present study is the first to report the essential oil composition and bioactivities of *W. glabra* from Tibet, China. The essential oil of *W. glabra* showed anti-influenza and anti-inflammatory effects, but no anti-complementary activity. The essential oil and the non-volatile extract should play different roles on the medical use of *W. glabra*. However, the composition of the essential oil *W. glabra* from Gangs Rinpoche Mountain (Trans-Himalaya Mountain Range in Tibet) were quite different from the reported *W. glabra* sample from Nepal. Thus, further comparison of the bioactivities and active compounds of *W. glabra* samples from north, south, and trans-Himalaya regions will be of great importance.

## Figures and Tables

**Table 1:** Chemical composition of the essential oil of *W. glabra* from Tibet.

No.	Compounds	% Area	RI ^1^
1	α-Pinene	0.3	944
2	Yomogi alcohol	1.4	996
3	Artemisia ketone	0.2	1066
4	Artemisia alcohol	0.5	1086
5	Tanacetone	0.2	1122
6	Lavandulol	0.4	1173
7	Terpinen-4-ol	0.2	1197
8	α-Terpineol	0.2	1212
9	β-Citronellol	0.8	1234
10	Bornyl acetate	0.2	1299
11	β-Caryophyllene	6.1	1445
12	β-Farnesene	1.0	1459
13	α-Himachalene	0.4	1493
14	α-Curcumene	0.5	1496
15	Citronellyl butanoate	1.0	1531
16	δ-Cadinene	0.8	1537
17	Geranyl butyrate	0.2	1564
18	Citronellyl iso-valerate	1.7	1579
19	Patchoulanol	0.4	1588
20	Spathulenol	8.2	1608
21	Caryophyllene oxide	5.2	1617
22	Isospathulenol	0.4	1661
23	α-Bisabolol	20.2	1706
24	Valeranone	11.8	1713
25	Hexahydrofarnesyl acetone	0.2	1848
26	Tonghaosu	0.2	1980
27	Chamazulene	9.9	2002
	Monoterpene hydrocarbons	0.3	
	Oxygenated monoterpenes	3.9	
	Sesquiterpene hydrocarbons	8.7	
	Oxygenated sesquiterpenes	59.1	
	Ketones	0.2	
	Ester	0.2	
	Total identified	72.4	
	Others	24.1	
	Total detected	96.5	

^1^ Retention Indices, relative to C8–C30 *n*-alkanes on the HP-5MS column.

**Table 2:** In vitro antiviral activity against H_3_N_2_ of *W. glabra* essential oil.

Samples	Concentrations (μg·mL^−1^)	Cytotoxicity (%)	TC_50_^a^ (μg·mL^−1^)	Inhibition Rate for CPE (%)	IC_50_^b^ (μg·mL^−1^)
Essential oil	200	50	252.0	/	88.8
	100	0		37.5	
	50	0		25	
	25	0		0	
	12.5	0		0	
Ribavirin	250	0		75	37.2
	125	0		75	

^a^ TC_50_ is 50% cytotoxic concentration. ^b^ IC_50_ is the concentration of the sample inducing 50% inhibition. “/” not determined because of the cytotoxicity at 200 μg·mL^−1^.
